# Supplementary material for: Study from microcosms and mesocosms reveals Escherichia coli removal in high rate algae ponds during domestic wastewater treatment is primarily caused by dark decay
Source: PLoS One. 2022 Mar 17;17(3):e0265576. doi: 10.1371/journal.pone.0265576 (PMC8929646; doi:10.1371/journal.pone.0265576)
Supplement: S1 Appendix — (PDF) [file pone.0265576.s001.pdf]

S1 *E. coli* strain used in the study: isolation, cultivation, and description

Strain isolation: A 'wild' type *E. coli* strain was isolated from positive Quanti-Tray wells previously loaded with samples from a pilot HRAP treating primary wastewater (Chambonniere et al., 2020). For this purpose, broth from Quanti-Tray positive wells were sampled and serially diluted in 5 $mg{\cdot}L^{-1}$ peptone water. The serial dilutions were spread-plated on Brain Heart agar and grown overnight. Single colonies with morphology consistent with *E. coli* colonies were subsequently plated and grown on Brain Heart agar. A strain thus isolated was confirmed as *E. coli* by plating on Eosin Methylene Blue agar (Huang et al., 1997) and used in subsequent experiments.

Strain cultivation: Pure *E. coli* stock cultures grown on Brain Heart agar were stored at 4°C and renewed every third month. Before each experiment, *E. coli* cells from pure colonies on plates were aseptically transferred into 25 mL of Brain Heart broth and grown overnight at 37°C. This liquid culture was then stored at 4°C until the day of the experiment. On the day of the experiment, the culture was centrifuged at 4,400 rpm for 10 minutes (Eppendorf® centrifuge 5702). The medium was immediately discarded and the cells were resuspended in 30 mL of reverse osmosis (RO) water. These solutions were used as inoculum in laboratory and bench assays.

Strain characterization: To provide DNA for PCR analysis, colonies of the bacterium were aseptically re-suspended in 1mL of sterile distilled water in 2mL DNA-free Eppendorf tubes. Tubes were vortexed for 1 min at full speed and the suspension was used to extract DNA using QIAamp BiOstic Bacteremia DNA Kit (Qiagen, Germany) according to the manufacturer's protocol.

DNA extracts were quantified using a NanoDrop (ThermoFisher) and by running 8 μL of eluted DNA on an agarose gel (1.2% (w/v) agarose in 1× Tris-acetate–EDTA buffer containing SyBrSafe) and visualizing the gel on a UVDoc HD6 (ThermoFisher).

PCR was carried out on the extracted DNA using universal primers for bacterial 16S (F27: AGAGTTTGATCCTGGCTCAG; 1492R: TACGGYTACCTTGTTACGACGAC), and EmeraldAmp GT PCR Master Mix (Takara RR310A). The PCR conditions were: an initial denaturation of 95 ºC - 3 min followed by 35 cycles of: [95ºC - 30 s, 50ºC - 30 s, 72ºC – 90 s], a hold at 72ºC – 5 min, and a hold at 10 ºC. Following PCR, 8 μl of reaction mix was visualised on an agarose gel as above.

Each PCR gave a product of the expected size (1.4kb). The product was purified by gel extraction and sequenced in both directions on a ABI3730 capillary sequencer at the Massey Genome Service.

The three sequences were identical (see below):

TGCAGTCGAACGGTAACAGGAAGAAGCTTGCTTCTTTGCTGACGAGTGGCG
GACGGGTGAGTAATGTCTGGGAAACTGCCTGATGGAGGGGGATAACTACTG
GAAACGGTAGCTAATACCGCATAACGTCGCAAGACCAAAGAGGGGGACCTT
CGGGCCTCTTGCCATCGGATGTGCCCAGATGGGATTAGCTAGTAGGTGGGG
TAACGGCTCACCTAGGCGACGATCCCTAGCTGGTCTGAGAGGATGACCAGC
CACACTGGAACTGAGACACGGTCCAGACTCCTACGGGAGGCAGCAGTGGGG
AATATTGCACAATGGGCGCAAGCCTGATGCAGCCATGCCGCGTGTATGAAG
AAGGCCTTCGGGTTGTAAAGTACTTTCAGCGGGGAGGAAGGGAGTAAAGTT
AATACCTTTGCTCATTGACGTTACCCGCAGAAGAAGCACCGGCTAACTCCGT
GCCAGCAGCCGCGGTAATACGGAGGGTGCAAGCGTTAATCGGAATTACTGG

GCGTAAAGCGCACGCAGGCGGTTTGTTAAGTCAGATGTGAAATCCCCGGGC
TCAACCTGGGAACTGCATCTGATACTGGCAAGCTTGAGTCTCGTAGAGGGG
GGTAGAATTCCAGGTGTAGCGGTGAAATGCGTAGAGATCTGGAGGAATACC
GGTGGCGAAGGCGGCCCCCTGGACGAAGACTGACGCTCAGGTGCGAAAGC
GTGGGGAGCAAACAGGATTAGATACCCTGGTAGTCCACGCCGTAAACGATG
TCGACTTGGAGGTTGTGCCCTTGAGGCGTGGCTTCCGGAGCTAACGCGTTAA
GTCGACCGCCTGGGGAGTACGGCCGCAAGGTTAAAACTCAAATGAATTGAC
GGGGGCCCGCACAAGCGGTGGAGCATGTGGTTTAATTCGATGCAACGCGAA
GAACCTTACCTGGTCTTGACATCCACGGAAGTTTTCAGAGATGAGAATGTGC
CTTCGGGAACCGTGAGACAGGTGCTGCATGGCTGTCGTCAGCTCGTGTTGTG
AAATGTTGGGTTAAGTCCCGCAACGAGCGCAACCCTTATCCTTTGTTGCCAG
CGGTCCGGCCGGGAACTCAAAGGAGACTGCCAGTGATAAACTGGAGGAAG
GTGGGGATGACGTCAAGTCATCATGGCCCTTACGACCAGGGCTACACACGT
GCTACAATGGCGCATACAAAGAGAAGCGACCTCGCGAGAGCAAGCGGACC
TCATAAAGTGCGTCGTAGTCCGGATTGGAGTCTGCAACTCGACTCCATGAA
GTCGGAATCGCTAGTAATCGTGGATCAGAATGCCACGGTGAATACGTTCCC
GGGCCTTGTACACACCGCCCGTCACACCATGGGAGTGGGTTGCAAAAGAAG
TAGGTAGCTTAACCTTCGGGAGGGCGCTTACCAC

A BLASTn search of GenBank (NCBI) confirmed that the sequence was 100% identical for more than *E. coli* strains across 99.9-100% of its length**.** The 100% match for the full sequence was achieved against *E. coli* strain 244-c etp 16S ribosomal RNA gene, partial sequence (accession # MN208157).

Chambonniere, P., Bronlund, J., Guieysse, B., 2020. *Escherichia coli* removal during domestic wastewater treatment in outdoor high rate algae ponds: Long-term

performance and mechanistic implications. Water Sci. Technol. 1–10. https://doi.org/10.2166/wst.2020.233

Huang, S.W., Chang, C.H., Tai, T.F., Chang, T.C., 1997. Comparison of the β-glucuronidase assay and the conventional method for identification of *Escherichia coli* on eosin-methylene blue agar. J. Food Prot. 60, 6–9. https://doi.org/10.4315/0362-028X-60.1.6
