# Supplementary material for: Study from microcosms and mesocosms reveals Escherichia coli removal in high rate algae ponds during domestic wastewater treatment is primarily caused by dark decay
Source: PLoS One. 2022 Mar 17;17(3):e0265576. doi: 10.1371/journal.pone.0265576 (PMC8929646; doi:10.1371/journal.pone.0265576)
Supplement: S2 Appendix — (PDF) [file pone.0265576.s002.pdf]

S2: Detailed laboratory assay protocols

Specific methods used during laboratory assays for the investigation of single *E. coli* decay mechanisms are detailed below.

Starvation and heat inactivation: E-flasks were filled with reverse osmosis (RO) water and equilibrated at the desired temperature before inoculation. The flasks were then inoculated and incubated in darkness at constant temperature (5 - 35°C) for up to 7 days.

Toxicity of algal metabolites: Aliquots of 100 mL of pilot HRAP culture were mixed with 25 mL of primary settled wastewater in 250 mL E-flasks (Palmerston North wastewater treatment plant, New Zealand) and incubated in the conditions described by Béchet et al. (2015). On the day of the experiment, 50 mL of these cultures were filtered through GF/C™ grade fiberglass (General Electric®) and added to 150 mL E-flasks that were then inoculated with *E. coli* and incubated in darkness (23 ± 2°C) up to 7 days. The filtrates were not autoclaved to avoid the deactivation of potentially toxic compounds.

This experiment was repeated using filtrates of pilot HRAP broth collected no more than one hour prior to the experiment on a sunny day at 5 P.M. (total sunlight energy received at 5 P.M. was 20.2 $MJ \cdot m^{-2}$, maximal hourly averaged sunlight intensity of 830.6 $W \cdot m^{-2}$), and incubated overnight.

Wastewater toxicity: *E. coli* cells were suspended in filtrated or centrifuged wastewater and incubated in darkness at 23 ± 2°C for up to 7 days (wastewater withdrawn downstream of primary settling, Palmerston North wastewater treatment plant, New Zealand).

Ammonia toxicity: E-flasks were filled with 50 mL of pH buffer (Table S2-1) and 1 mL of ammonium chloride solution (3.7 mM or 109 mM) to reach a final total ammoniacal-nitrogen concentration of either 0.1 (representative of HRAP broth levels) or 30 mg $N\text{-}NH_3.L^{-1}$ (representative of primary settled domestic wastewater levels) at pH 8 to 10.Reverse osmosis water was used as control. These flasks were incubated in darkness at constant temperature ranging from 10 to 32°C for 3 to 7 hours respectively to decreasing temperature. Ammoniacal-nitrogen concentration was verified using an Orion AQUAfast AQ3700 colorimeter based on the manufacturer instructions (Thermofisher, USA).

Alkaline-pH induced toxicity: E-flasks were filled with pH-buffers (Table S2-1) equilibrated at the desired temperature (5 - 35°C). The flasks were then inoculated and incubated in darkness at constant temperature (Contherm incubator, Thermofisher, USA) for 2 hours to 2 days respectively to decreasing temperature. These experiments were repeated in open beakers exposed to direct sunlight.

Direct sunlight damage: *E. coli* cells were suspended in RO water and exposed to direct sunlight on the laboratory rooftop (Palmerston North, New Zealand) for 2 to 4 hours. This experiment was repeated in pH 10 buffer (Table S2-1). Dark controls were conducted in 150 mL E-flasks covered with foil and incubated outdoor.

Exogenous photo-oxidation: *E. coli* cells were suspended in wastewater or HRAP filtrates and exposed to direct sunlight for 2 hours. This experiment was repeated in pH 10 buffer (Table S2-1). Dark controls were also conducted.

Table S2-1. pH buffers recipe(Dawson et al.,

1986)

**Buffer formula**	**expected pH (25°C)**
20 mL 0.2 M KH_2PO4	7.0
30 mL 0.2 M Na_2HPO_4	
2.5 mL 0.2 M KH_2PO4	8.0
47.5 mL 0.2 M Na_2HPO_4	
45 mL 0.1 M $NaHCO_3$	9.2
5 mL 0.1 M Na_2CO_3	
25 ml 0.1 M $NaHCO_3$	9.9
25 mL 0.1 M Na_2CO_3	
5 mL 0.1 M $NaHCO_3$	10.8
45 mL 0.1 M Na_2CO_3	

Béchet, Q., Plouviez, M., Chambonnière, P., Guieysse, B., 2017. Environmental impacts of full-scale algae cultivation, Microalgae-Based Biofuels and Bioproducts: From Feedstock Cultivation to End-Products. https://doi.org/10.1016/B978-0-08-101023-5.00021-2

Dawson, R.M.C., Elliott, D.C., Elliott, W.H., Jones, K.M., 1986. Data for biochemical research, 3rd ed. Oxford Science Publ.
