# Supplementary material for: Study from microcosms and mesocosms reveals Escherichia coli removal in high rate algae ponds during domestic wastewater treatment is primarily caused by dark decay
Source: PLoS One. 2022 Mar 17;17(3):e0265576. doi: 10.1371/journal.pone.0265576 (PMC8929646; doi:10.1371/journal.pone.0265576)
Supplement: S3 Appendix — (PDF) [file pone.0265576.s003.pdf]

# S3 Uncertainty of cell count obtained from pour-plate method

The uncertainty associated with pour-plate cell counts was assessed by assuming no decay was taking place in microcosms incubated in the dark and supplied RO water or neutral pH buffer (see S2). Hence, the relative difference ($diff$) in *E. coli* cell counts between the initial measurement and each following measurement was calculated for every such reactor as:

$$diff = \frac{C_0 - C}{C_0} \qquad \textbf{(S3-1)}$$

where $C_0$ is the *E. coli* cell count measured at the beginning of the experiment (CFU·mL$^{-1}$) and $C$ is the *E. coli* cell count measured at the time for which the deviation is calculated (CFU·mL$^{-1}$). The same analysis was performed over the log transformed counts. The average value of $diff$ is expected to be zero, and the standard deviation calculated from $diff$ statistical distribution is indicative of the measurement error during counting (USEPA, 2001). The distribution of the relative differences calculated over the raw and log-transformed counts are shown in Fig S3-1. As can be seen, both distributions follow a normal law, evidencing that the spread of the deviations is likely due to random errors as hypothesized. The relative standard error of the raw cell counts and the log-transformed cell counts were thus estimated to be ± 84 % and ± 4.0% respectively.

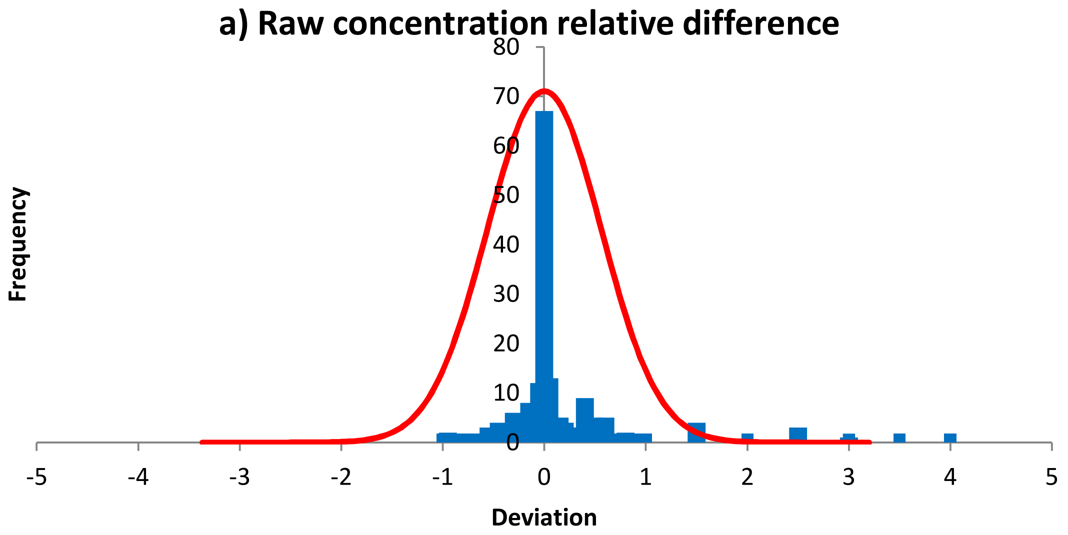


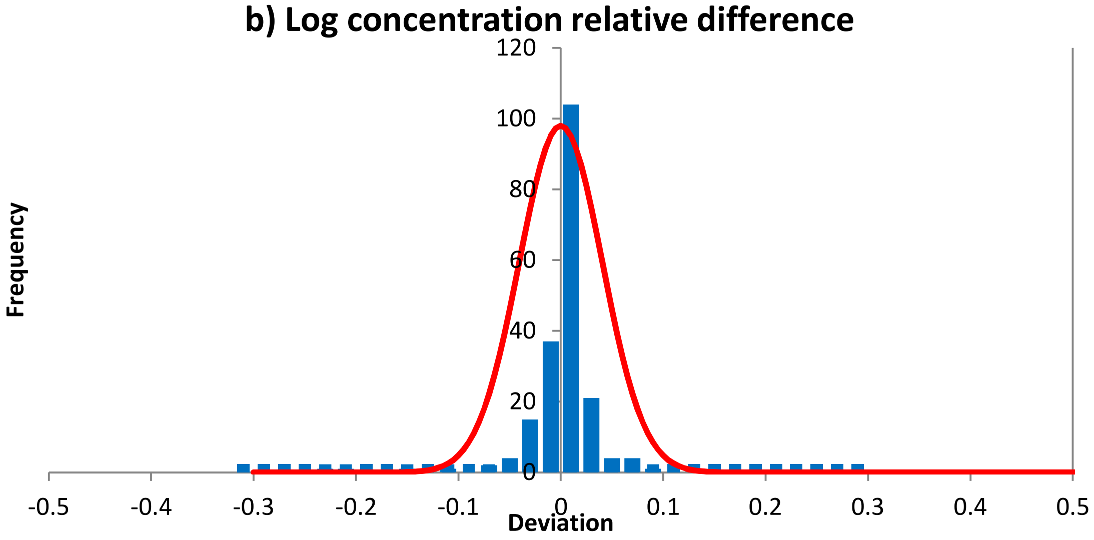


**Fig. S3-1. Distribution of the measured deviation in *E. coli* cell counts from initial counts in reactors showing no significant changes in viable cell counts over time.** The red curves represent the normal law $\mathcal{N}(0, \sigma^2)$ where $\sigma^2$ is the standard deviation calculated for the sample of calculated $dev$ values.

USEPA, 2001. Risk Assessment Guidance for Superfund (RAGS) Volume III - Part A: Process for Conducting Probabilistic Risk Assessment, Appendix B, Office of Emergency and Remedial Response U.S. Environmental Protection Agency.
