# Supplementary material for: Study from microcosms and mesocosms reveals Escherichia coli removal in high rate algae ponds during domestic wastewater treatment is primarily caused by dark decay
Source: PLoS One. 2022 Mar 17;17(3):e0265576. doi: 10.1371/journal.pone.0265576 (PMC8929646; doi:10.1371/journal.pone.0265576)
Supplement: S4 Appendix — (PDF) [file pone.0265576.s004.pdf]

S4 Calculation of first order decay rate and associated uncertainty during laboratory assays

Calculation of first order decay rates

Typically, three or more detectable cell counts were obtained from a single reactor monitored during laboratory assay, enabling to perform a linear regression on the natural logarithm of these cell counts over the time of sampling. The slope of the regression was used to quantify the *E. coli* decay rate for the given conditions. Shoulder log-linear kinetics (Blaustein et al., 2013; Silverman and Nelson, 2016) for *E. coli* decay were not evidenced during laboratory scale experiments, although these kinetics could still apply to shorter-term (< 3h) experiments investigating the kinetics of exogenous photo-oxidation (see specific discussion in S12).

If only two points were available, first order decay rate was calculated as $\frac{\ln\left(\frac{C_0}{C}\right)}{t}$ where C_0 and C are the values of the cell counts at the start and latest suitable times of sampling, respectively, and t is the time of the latest such sampling events.

A linear regression over the entire data set recorded was not meaningful under natural sunlight exposure due to the temporal variability of natural sunlight intensity and temperature. Consequently, a value of the decay rate was calculated between each consecutive cell count recorded according to the same formula as used when only two data points were available.

Uncertainty of first order decay rates

When three or more cell counts were available for a given reactor under stable conditions, the uncertainty of *E. coli* decay rates was determined based on the standard error of the regression coefficient as computed by the function *fitlm* of Matlab® R2019a (Mathworks Inc., Natick, MA, USA).

When *E. coli* decay rate was calculated from two consecutive cell counts, the uncertainty was determined based on the standard error of the difference of the two cell counts. The relative standard error of a log-transformed cell count was determined to be 4% (see S3). Therefore, let C_0 and C_1 be two consecutive cell counts during an interval Δt, the standard error of the first order decay rate k_d $(= (\ln(C_0) - \ln(C_1))/\Delta t)$ was calculated as:

$$\sigma_{k_d} = \frac{\sqrt{(0.04)^2 \cdot (\ln(C_0))^2 + (0.04)^2 \cdot (\ln(C_1))^2}}{\Delta t} = 0.04 \cdot \frac{\sqrt{(\ln(C_0))^2 + (\ln(C_1))^2}}{\Delta t}$$

As a consequence of this formula, measurement error associated to *E. coli* decay rate was inflated during short experiments. This was particularly the case during the investigation of sunlight mediated mechanisms.

Despite high measurement uncertainty, significantly positive decay rates were evidenced during this study. This evidences that targeted mechanisms not found significant based on measurement error are also unlikely to be significant in comparison with other mechanisms evidenced in this study.

Blaustein, R.A., Pachepsky, Y., Hill, R.L., Shelton, D.R., Whelan, G., 2013. *Escherichia coli* survival in waters: Temperature dependence. Water Res. 47, 569–578. https://doi.org/10.1016/j.watres.2012.10.027

Silverman, A.I., Nelson, K.L., 2016. Modeling the endogenous sunlight inactivation rates of laboratory strain and wastewater *E. coli* and enterococci using biological

weighting functions. Environ. Sci. Technol. 50, 12292–12301.
https://doi.org/10.1021/acs.est.6b03721
