# Supplementary material for: Study from microcosms and mesocosms reveals Escherichia coli removal in high rate algae ponds during domestic wastewater treatment is primarily caused by dark decay
Source: PLoS One. 2022 Mar 17;17(3):e0265576. doi: 10.1371/journal.pone.0265576 (PMC8929646; doi:10.1371/journal.pone.0265576)
Supplement: S5 Appendix — (PDF) [file pone.0265576.s005.pdf]

S5 Description of Monte Carlo method for the determination of fitted model parameters uncertainty

Uncertainty in experimental measurements caused uncertainties in the determination of model parameters during model parameterization. These uncertainties were estimated by randomly varying experimental inputs within their range of uncertainty assuming normal or log-normal distribution (Table S5-1).

Table S5-1. Variables and parameters used in Equation 4: value and uncertainty used for Monte-Carlo simulation

Variable/Parameter	Base value	95% confidence interval	Rationale of uncertainty range
E. coli **cell count in the bench reactor** ($\boldsymbol{C}$)	As measured	± 20 % on the log10 transformed measurement	Conservative estimates adding MPN function uncertainty and measurement error
**pH** ($\boldsymbol{pH}$)	As measured	± 0.01	Sensor specification
**Temperature** ($\boldsymbol{T}$)	As measured	± 0.1°C	Sensor specification
**Sunlight intensity** ($\boldsymbol{Hs}$)	Data provided by the National Institute of Water and Atmospheric Research Ltd	± 10 % of provided data	Conservative estimate
**Depth** ($\boldsymbol{d}$)	0.25 m	± 0.03 m	Conservative measurement error
**Light attenuation coefficient** ($\boldsymbol{\sigma}$)	55 – 70 m^{-1} (see main manuscript)	± 10 m^{-1}	Based on measurement uncertainty analysis by Béchet et al. (2015)

Equation 4 (main manuscript) was fitted to bench experimental data for each randomly generated set of model inputs by optimizing model fitted parameters as performed during model parameterization (main manuscript). This operation was repeated 1,000 times. The distribution of fitted parameters thus obtained is shown below. As can be seen, this distribution was not necessarily normal (e.g. α, Fig S5-1).

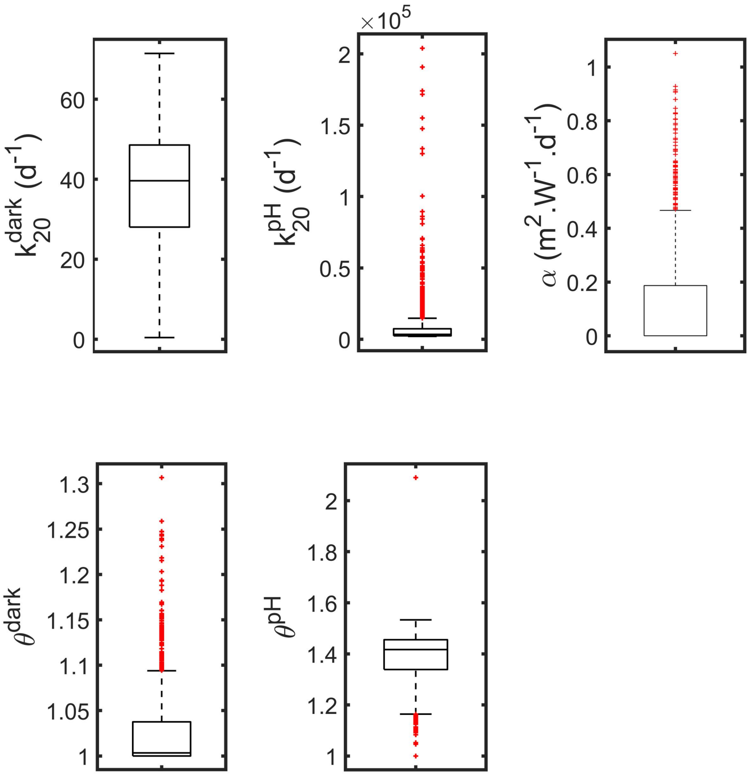

Fig. S5-1. Distribution of model fitted parameters as calculated using Monte Carlo analysis. Boxplots represent the 5, 25, 50, 75, and 95 percentiles of the distributions, and red dots the outliers.

Béchet, Q., Chambonnière, P., Shilton, A., Guizard, G., Guieysse, B., 2015. Algal productivity modeling: A step toward accurate assessments of full-scale algal cultivation. Biotechnol. Bioeng. 112. https://doi.org/10.1002/bit.25517
