# Supplementary material for: Study from microcosms and mesocosms reveals Escherichia coli removal in high rate algae ponds during domestic wastewater treatment is primarily caused by dark decay
Source: PLoS One. 2022 Mar 17;17(3):e0265576. doi: 10.1371/journal.pone.0265576 (PMC8929646; doi:10.1371/journal.pone.0265576)
Supplement: S6 Appendix — (PDF) [file pone.0265576.s006.pdf]

S6 Uncertainty associated with the relative contribution of studied mechanism to *E. coli* decay during bench assays

The confidence interval associated with the relative contribution of each considered mechanism to the overall *E. coli* decay was assessed using a Monte Carlo simulation. This simulation consisted in computing relative contributions when randomly varying model inputs and model fitted parameters within their respective 95% uncertainty intervals. The experimental uncertainties taken into account are listed in Table S5-1. The uncertainties associated with the fitted model parameters (i.e. k_{20}^{dark}, θ^{dark}, k_{20}^{pH}, θ^{pH}, and α) were computed based on the data generated during our prior Monte Carlo analysis (Table 2 of main manuscript): for each fitted parameter, random values were generated by aiming at creating distributions similar to the distribution obtained during Monte Carlo simulation for uncertainty analysis. This was achieved as explained below.

- The distribution of k_{20}^{dark} was assimilated to a type I extreme value distribution of parameters $\mu = 44.5$ (location parameter) and $\sigma = 12.4$ (scale parameter), noted $\widehat{k_{20}^{dark}} = GEV(44.5{,}12.4{,}0)$.
- The distribution of θ^{pH} was likewise assimilated to the distribution $\widehat{\theta^{pH}} = GEV(1.43{,}6.56 \cdot 10^{-2}, 0)$
- For approximately half of the data calculated during uncertainty analysis, θ^{dark} was found equal to 1 (N = 1072). When θ^{dark} was superior to one, it was found to be mathematically linked to k_{20}^{dark} as shown in Fig S6-1 a). Random values of

θ^{dark} were therefore generated as $\widehat{\theta^{dark}} = \max\left(1; \mathcal{B}(0.5)\cdot 1 + \left(1 - \mathcal{B}(0.5)\right)\cdot\left(1.2356\cdot \widehat{k_{20}^{dark}}^{\,-0.051} + \mathcal{N}(0,0.0202)\right)\right)$[1],

- Likewise, based on the data computed during uncertainty analysis, k_{20}^{pH} was mathematically linked to θ^{pH}, as shown in Figure S6-1 b). Random values of k_{20}^{pH} were thus calculated as $\widehat{k_{20}^{pH}} = 10^{-3.7302\cdot\widehat{\theta^{pH}}+8.8475+\mathcal{N}(0,0.109)^{2}}$.
- Based on the distribution generated during the first Monte Carlo analysis, random values of α were finally generated as $\hat{\alpha} = \mathcal{B}(0.5)\cdot Exp(0.2388)$ where $Exp(\mu)$ is the exponential probability distribution function of parameter μ.The Bernoulli distribution was used as approximately half of the values calculated for α during uncertainty analysis were 0 (N = 950).

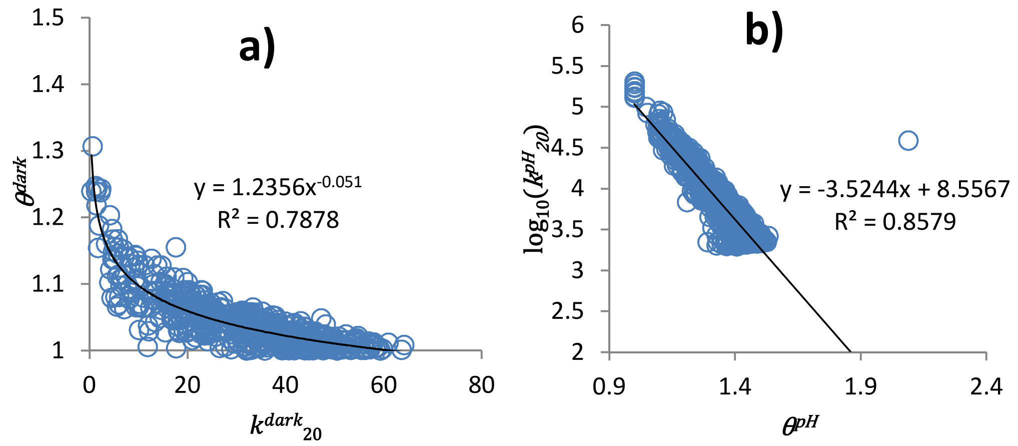

Fig. S6-1. θ^{dark} according to k_{20}^{dark} (a), and $log_{10}(k_{20}^{pH})$ according to θ^{pH} (b) for the data sets computed during uncertainty analysis. The mathematical laws found to

[1] $\mathcal{B}(p)$ represents a Bernoulli distribution of parameter p. The same value was used for the two laws $\mathcal{B}(0.5)$ visible in the expression of $\widehat{\theta^{dark}}$ in each generated value.

[2] The normal law used in the two latter distributions is based on the mean and standard deviation of the residuals between observed values and values modelled as shown in Figure A6-1 (the residuals were not strictly normally distributed but acceptable).

best fit these distributions were fitted and are shown with Equation and correlation coefficient. Data for which $\theta^{dark} = 1$ (N = 1072) were excluded from (a)

2,000 datasets of randomly generated model inputs were thus created. Datasets including values of fitted parameters outside of their respective 95 percentile were removed from the analysis. The final Monte Carlo analysis was therefore performed over 1,322 randomly generated data sets.

Comparison between the distributions of the randomly generated values for the five fitted parameters and the distributions generated during the initial Monte Carlo analysis is shown on Fig S6-2 for both data sets limited to the 5-95 percentiles presented in Table 3 of the main manuscript. The differences observed are explained by the fact the initial distributions were not following clear statistical/mathematical laws. However, the new distributions remain representative and the data generated could be used for Monte Carlo simulations.

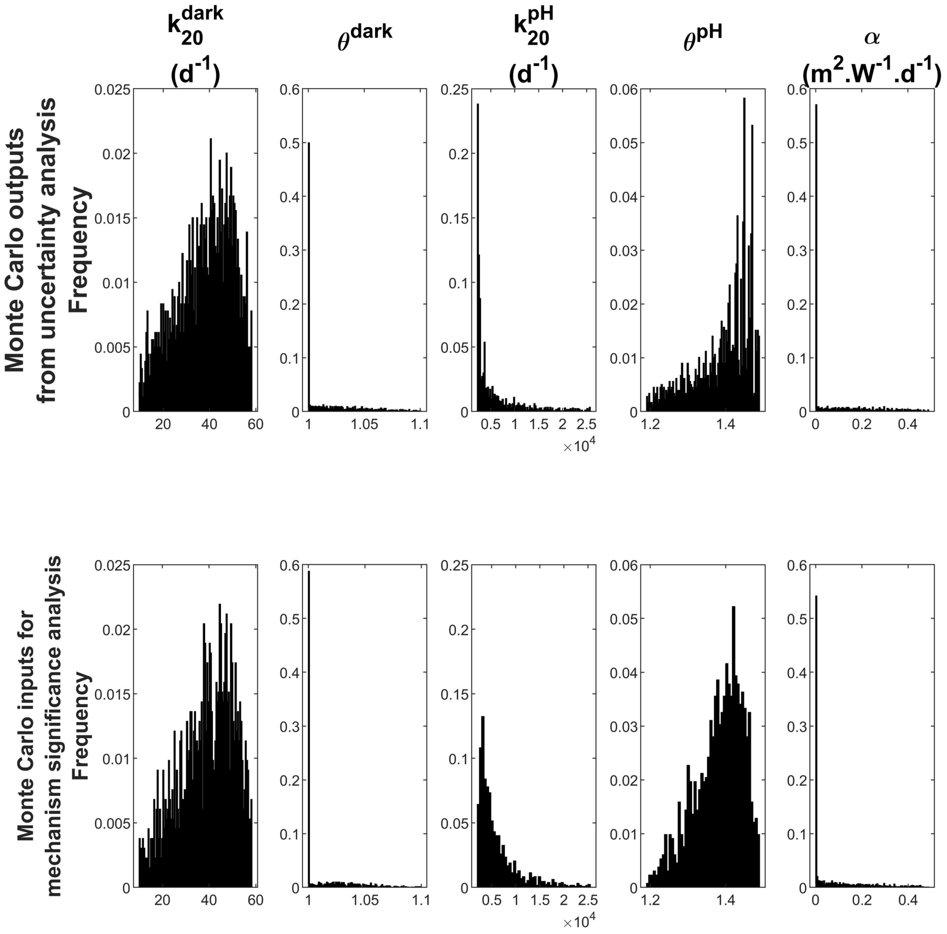

Fig. S6-2. Distributions (in relative frequency) of the fitting parameters (k_{20}^{dark}, θ^{dark}, k_{20}^{pH}, θ^{pH}, and α from left to right) obtained from Monte Carlo uncertainty analysis (top) and used for Monte Carlo analysis of mechanism significance (bottom)
