# Supplementary material for: Study from microcosms and mesocosms reveals Escherichia coli removal in high rate algae ponds during domestic wastewater treatment is primarily caused by dark decay
Source: PLoS One. 2022 Mar 17;17(3):e0265576. doi: 10.1371/journal.pone.0265576 (PMC8929646; doi:10.1371/journal.pone.0265576)
Supplement: S7 Appendix — (PDF) [file pone.0265576.s007.pdf]

S7 *E. coli* starvation and heat inactivation during laboratory assays

The distribution of experimental decay rates recorded in dark microcosms filled with RO water or neutral pH buffer (6.5 < pH < 7.5) and controlled temperature (5 – 35°C) is shown on Fig S7-1. As can be seen, the distribution can be considered as normal and spread around zero (average of 0.67 ± 3.76 d^{-1}, N = 30, hypothesis not rejected by a one-sample t-test at the 95% confidence level, p = 0.340).

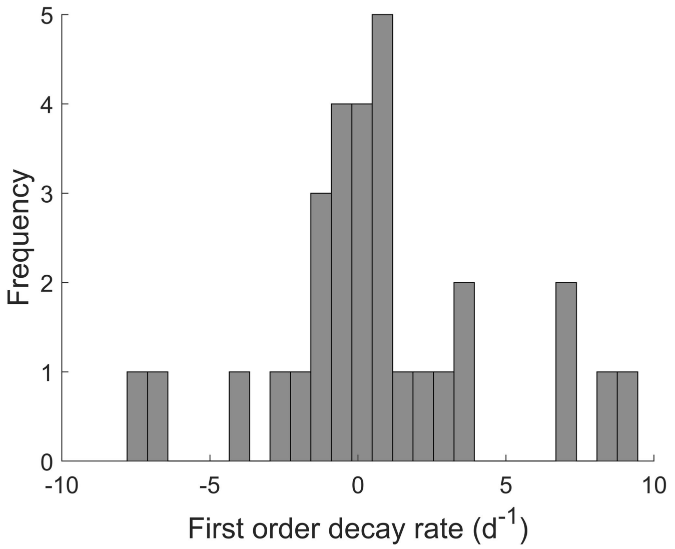

Fig. S7-1. Distribution of *E. coli* first order deacay rate calculated from laboratory experiments performed in dark microcosms filled with RO water or neutral pH buffer

While temperature remained constant throughout the experiment for a given dark microcosm, it varied from 5 to 35°C between different experiments, which may have influenced the distribution of decay rates measured. The decay rates recorded in each

reactors included in this study are displayed in Fig S7-2 according to their incubation temperature (± 2°C). As can be seen, there was no clear relationship between decay rates and temperature although the spread in decay rates increased with increasing temperature. This was associated to the increase in measurements error due to shorter experiment durations at higher temperature.

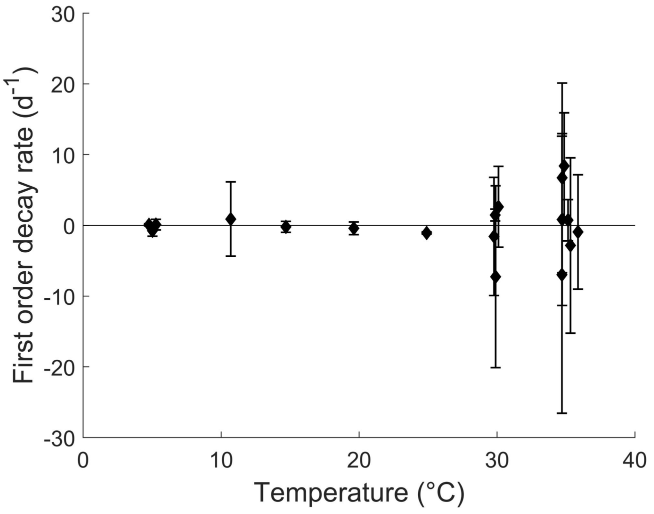

Fig. S7-2. ***E. coli*** **decay rates in dark controls during temperature-controlled experiments according to the incubation temperature.** Error bars show the standard error the measurement.

As the variability of *E. coli* decay rates recorded in dark microcosms filled with RO water or at neutral pH buffer could not be explained by variations of temperature, this variability was likely caused by measurement uncertainty (e.g. all decay rate values higher than 0.9 d^{-1} or lower than -0.9 d^{-1} were computed over short-term experiments < 24h, associated to 95 % confidence interval higher than ±2.8 d^{-1} based on the established 4 % standard error for measured log-transformed *E. coli* cell counts using pour plate method, S3 Appendix, and assuming initial cell counts of $50 \cdot 10^6$ CFU·ml^{-1}).
