# Supplementary material for: Study from microcosms and mesocosms reveals Escherichia coli removal in high rate algae ponds during domestic wastewater treatment is primarily caused by dark decay
Source: PLoS One. 2022 Mar 17;17(3):e0265576. doi: 10.1371/journal.pone.0265576 (PMC8929646; doi:10.1371/journal.pone.0265576)
Supplement: S8 Appendix — (PDF) [file pone.0265576.s008.pdf]

S8 Ammonia toxicity to *E. coli* during laboratory assays

The impact of NH_3 concentration on *E. coli* decay at varying pH is shown on Fig S8-1. The values of the decay rates shown were normalized at 20˚C (based on Equation 2 developed in the main manuscript) to compare results obtained at different temperatures. No significant decay was found at pH $\leq$ 9, regardless of the amount of NH_4Cl added. Significant decay was recorded at pH 10. The impacts of temperature and NH_3 concentration on *E. coli* decay was specifically studied at pH 10 (Fig S8-2). At this pH, *E. coli* decay exponentially increased with temperature at all NH_3 concentrations tested, but *E. coli* decay at pH 10 was not significantly enhanced by the addition of ammonium salt, regardless of the temperature and NH_4Cl concentration. NH_3 was therefore concluded not to impact *E. coli* decay at typical NH_3 concentration, pH, and temperatures experienced in HRAPs.

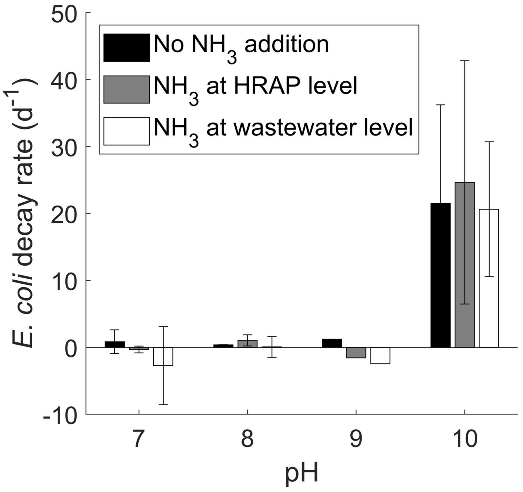

Fig. S8-1. Effect of NH_3 salt addition on *E. coli* removal performances at different pH normalized for 20°C. The data presented is the average of the value measured in given conditions; error bars displays the standard deviation within the measurement.

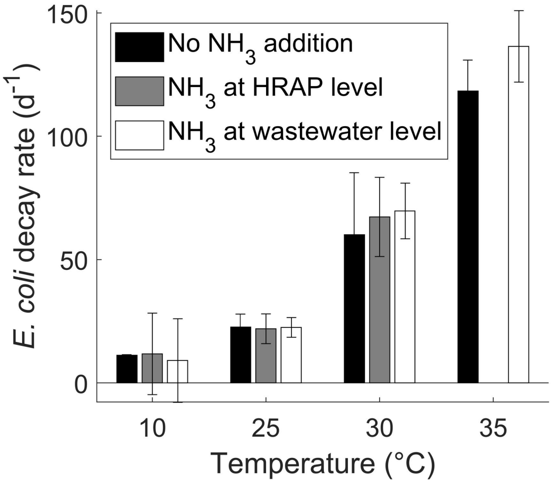

Fig. S8-2. Effect of NH_3 salt addition on *E. coli* decay at pH 10 for different temperatures. Error bars displays the standard error from measurement
