# Supplementary material for: Study from microcosms and mesocosms reveals Escherichia coli removal in high rate algae ponds during domestic wastewater treatment is primarily caused by dark decay
Source: PLoS One. 2022 Mar 17;17(3):e0265576. doi: 10.1371/journal.pone.0265576 (PMC8929646; doi:10.1371/journal.pone.0265576)
Supplement: S9 Appendix — (PDF) [file pone.0265576.s009.pdf]

S9 Alkaline pH induced toxicity to *E. coli* during laboratory assays

Example of the variation of *E. coli* decay measured during laboratory assays according to pH at constant temperature (30°C) and according to temperature at constant pH (10) are shown on Fig S9-1 and S9-2, respectively.

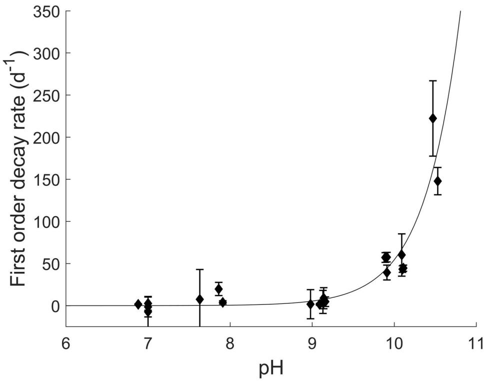

Fig. S9-1. Influence of pH on *E. coli* decay rate at 30°C in laboratory microcosms (plain line represents exponential model best fit, $y = 5.449 \cdot 10^5 \cdot 10^{x-14}$, R^2 = 0.8789, N = 20). Error bars show the standard error of the measured data

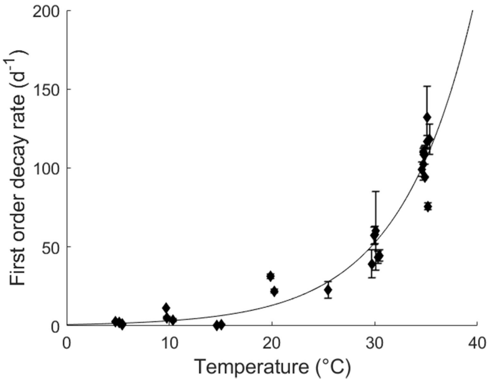

Fig. S9-2. Influence of temperature on *E. coli* decay rate at pH 10 in laboratory microcosms (plain line represents exponential model best fit, $y = 0.7925 \cdot e^{0.14 \cdot x}$, R^2 = 0.945, N = 27). Error bars show the standard error of the measured data

The values obtained from the linear regressions of *E. coli* decay rates measured at constant temperature and 10^{pH-14} ($a(T)$) are shown in Fig S9-3 and the results from the linear regressions are described in Table S9-1.

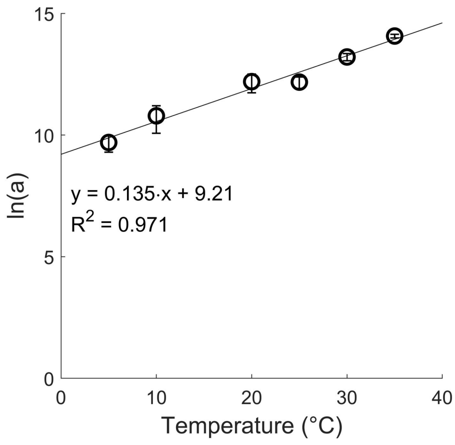

Fig. S9-3. Variations of the log-transformed *E. coli* decay at pH 14 ($\boldsymbol{a(T)}$) according to temperature during laboratory assays. The plain line represents the linear model associated (N = 6, R^2 = 0.971, $ln(a(T)) = (0.135 \pm 0.0116) \cdot T + 9.21 \pm 0.270$, p = $3.08 \cdot 10^{-4}$ and $4.42 \cdot 10^{-6}$ respectively). Error bars show the standard error of the fit performed to obtain $a(T)$ values.

Table S9-1. Results from the linear regression between [OH^-] and *E. coli* decay rate for a given temperature

**T (°C)**	**5**	**10**	**20**	**25**	**30**	**35**
$\boldsymbol{a(T)}$ **(L.mol^{-1}.d^{-1})**	$1.62 \cdot 10^4$	$4.85 \cdot 10^4$	$1.98 \cdot 10^5$	$1.94 \cdot 10^5$	$5.45 \cdot 10^5$	$1,29 \cdot 10^6$
**[95% confidence bounds]**	$[1.09;2.15] \cdot 10^4$	$[2.37;7.33] \cdot 10^4$	$[1.25;2.70,] \cdot 10^5$	$[1.49;2.38] \cdot 10^5$	$[4.65;6.25] \cdot 10^5$	$[1.19;1.39] \cdot 10^6$
$\boldsymbol{R^2}$	0.7086	0.6660	0.7042	0.9324	0.8789	0.9452
**N**	13	6	7	7	20	23
**RMSE**	0.535	2.33	5.91	2.21	19.8	12.3
