# Supplementary material for: Study from microcosms and mesocosms reveals Escherichia coli removal in high rate algae ponds during domestic wastewater treatment is primarily caused by dark decay
Source: PLoS One. 2022 Mar 17;17(3):e0265576. doi: 10.1371/journal.pone.0265576 (PMC8929646; doi:10.1371/journal.pone.0265576)
Supplement: S10 Appendix — (PDF) [file pone.0265576.s010.pdf]

# S10 Decay of laboratory versus wildtype *E. coli*

In order to evidence if a “domestic” strain ATCC® 10536™ and two isolated (S1) “wild” strains (named #1 and #2) exhibited similar survival abilities, the decay of these strains was monitored under harmful conditions previously identified in laboratory assays (see main manuscript). Two comparative experiments were performed, one exposing the bacteria to sunlight, and one recording the decay of these bacteria at high pH in the dark.

A rooftop experiment in RO water was performed following procedures described in the main manuscript. The experiment was carried out in Palmerston North, New Zealand (40°23’15”S 175°37’08”E) on the 7 of December 2016. This day was mostly overcast but significant sunlight energy was recorded at ground level (Fig S10-1). Two 100 mL open beaker exposing bacteria to sunlight induced damage were prepared for each strain, and one foil-covered E-flask was used as dark control. The bacteria were exposed to sunlight from 12:30 to 15:30. Samplings were performed at the start and end of exposition.

A typical pH experiment in the dark was conducted at ambient temperature (23 ± 2 °C) following the procedure described in the main manuscript. The three *E. coli* strains were each inoculated in one E-flask filled with 50 mL MQ water and one E-flask filled with pH 10 buffer. The reactors were sampled twice, once just after inoculation and once after a 4h incubation period.

**Sunlight mediated damage:** The sunlight energy received at ground level on 7 December 2016 in Palmerston North[3] is shown in Fig S10-1.

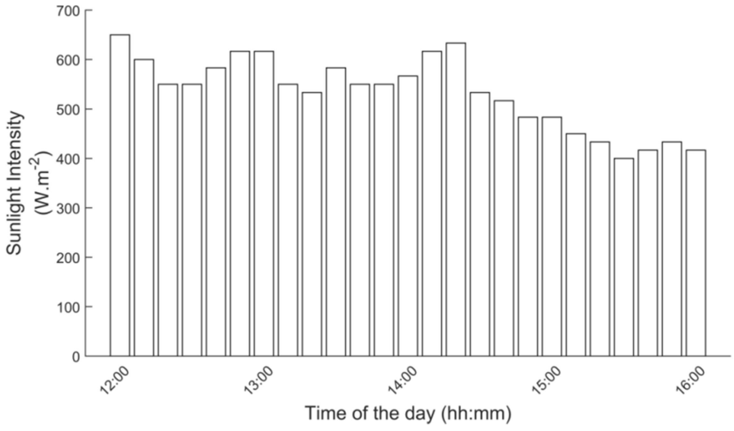


**Fig. S10-1. Sunlight intensity recorded on the 07/12/2016**

*E. coli* cell counts are presented in Fig S10-2 and S10-3.

While the initial cell counts are similar for all 3 strains, the decay coefficient of the 'laboratory' strain ATCC® 10536™ was significantly higher than the decay coefficients of the wild strains exposed to the same conditions of sunlight or elevated pH. For all strains, decay was insignificant under darkness at neutral pH (not shown).

[3] Data obtained from National Institute of Water and Atmospheric Research Ltd. database (Palmerston North, station ID 21963)

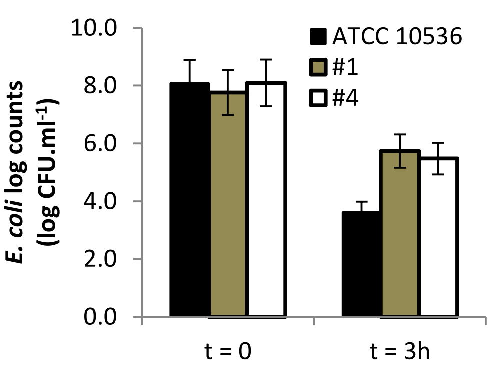


**Fig. S10-2. Log counts of *E. coli* strains (ATCC® 10536™ and wildtype) prior and after exposition to sunlight.** The error bars show measurement standard error.

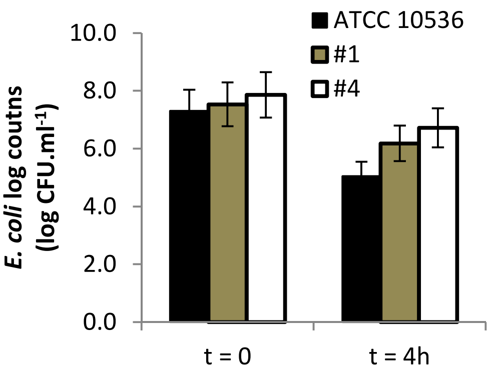


**Fig. S10-3. Log counts of *E. coli* strains (ATCC® 10536™ and wildtypes) prior and after exposition to pH 10.** The error bars show measurement standard error.

The higher sensitivity of the laboratory strain was therefore confirmed for two removal mechanisms. This experiment validates the need to conduct experiments using wild strains of *E. coli,* as suggested by Fisher and Nelson (2014) and Silverman and Nelson (2016). Experiments presented in the main manuscript thesis were performed using *E. coli* #1 strain. Interestingly, no significant difference in cell viability was found between both wild strains.


Fisher, M.B., Nelson, K.L., 2014. Inactivation of *Escherichia coli* by polychromatic simulated sunlight: Evidence for and implications of a fenton mechanism involving iron, hydrogen peroxide, and superoxide. Appl. Environ. Microbiol. 80, 935–942. https://doi.org/10.1128/AEM.02419-13

Silverman, A.I., Nelson, K.L., 2016. Modeling the endogenous sunlight inactivation rates of laboratory strain and wastewater *E. coli* and enterococci using biological weighting functions. Environ. Sci. Technol. 50, 12292–12301. https://doi.org/10.1021/acs.est.6b03721
