# Supplementary material for: Study from microcosms and mesocosms reveals Escherichia coli removal in high rate algae ponds during domestic wastewater treatment is primarily caused by dark decay
Source: PLoS One. 2022 Mar 17;17(3):e0265576. doi: 10.1371/journal.pone.0265576 (PMC8929646; doi:10.1371/journal.pone.0265576)
Supplement: S11 Appendix — (PDF) [file pone.0265576.s011.pdf]

S11 Interaction between elevated pH and sunlight mediated *E. coli* decay

E. coli decay under sunlight was significantly faster at high pH than neutral pH (Fig S11-1). In several instances at pH 10 and high sunlight intensities, rates could not be calculated because *E. coli* decay was too rapid for viable *E. coli* cells to be found at the second sampling point.

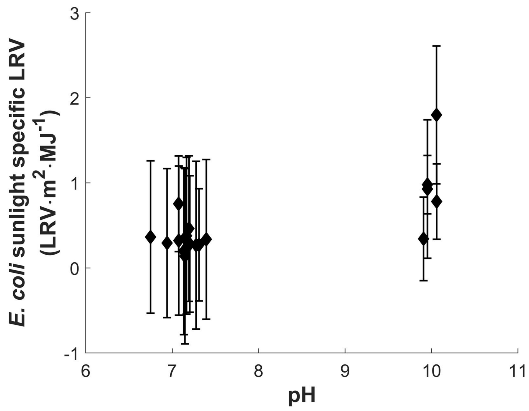

Fig. S11-1. Influence of pH on *E. coli* LRV normalized for sunlight exposure during laboratory assays. Error bars show the mesurement standard error.

Because high-pH induced toxicity was evidenced under darkness, it was unclear if the combined action of high-pH and sunlight was synergetic or merely additive. To investigate this, the difference between the 'light rate' recorded at pH 10 and the theoretical 'pH-induced' rate calculated at the corresponding pH and temperature was compared to 'light decay' recorded at neutral pH (theoretically only caused by direct photo-damage). The potential pH synergetic effect was computed as $(\tilde{k}_{light}^{10} - k_{light}^{7})/k_{light}^{7}$ where $\tilde{k}_{light}^{10}$ is the difference between the experimental decay rate measured at pH 10 under sunlight and the theoretical decay rate at pH 10 in darkness at

the reported temperature (see Equation 3 in main manuscript), and k_{light}^{7} is the experimental decay rate measured under the same sunlight conditions at neutral pH.

Synergetic effects of 523 %, 62 %, 77 %, 242 %, and -138 % were found in order of increasing sunlight intensity. Despite the large variation in the magnitude of the impact, the results suggested a positive interaction between sunlight and pH rather than solely an addition of toxicity effects. Unfortunately, measurement uncertainty (arising from the counting method and variability in the environmental conditions) limited the use of statistics for a more precise assessment.

Finally, results from tests performed in HRAP filtrates buffered at pH 10 to investigate potential synergetic effect of pH with exogenous photo-oxidation did not evidence any clear synergy though only two comparable decay rate measurements could be retrieved: one of the test yielded a 199% relative increase of *E. coli* decay rate while the second measurement evidenced a relative decrease of *E. coli* decay rate by 28.5% at pH 10 compared with neutral pH in HRAP filtrates under natural sunlight.

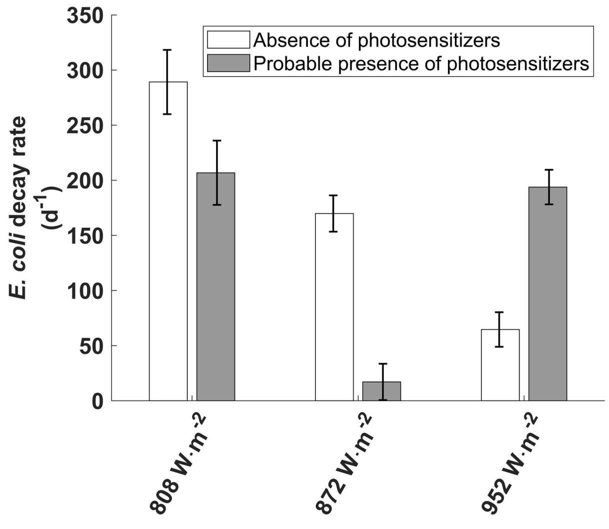

Fig. S11-2. *E. coli* decay rate in RO water or filtrates from HRAP broth under natural sunlight at pH 10 (laboratory assays). Error bars show the standard error of the measured decay rates.
