# Supplementary material for: Study from microcosms and mesocosms reveals Escherichia coli removal in high rate algae ponds during domestic wastewater treatment is primarily caused by dark decay
Source: PLoS One. 2022 Mar 17;17(3):e0265576. doi: 10.1371/journal.pone.0265576 (PMC8929646; doi:10.1371/journal.pone.0265576)
Supplement: S12 Appendix — (PDF) [file pone.0265576.s012.pdf]

S12 Impact of photosensitizers on *E. coli* decay under sunlight during laboratory assays

An example of absorption spectrum of wastewater and HRAP filtrates (sampled from the set-up used in the present study) is shown in Fig S12-1.

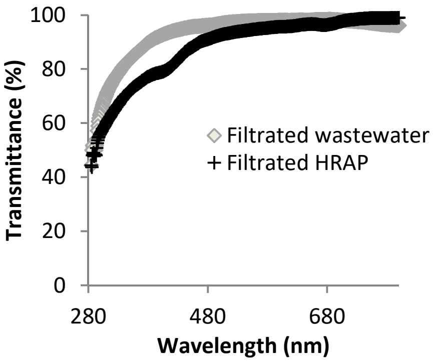

Fig. S12-1. Transmittance through one centimetre of filtrated wastewater and HRAP broth (measured on 13/04/2017)

Existence of shoulder-first order decay kinetics for exogenous photo-oxidation:
Due to limitations of the experimental design, reactors were not exposed to sunlight for more than 2 hours during laboratory assays measuring the impact of exogenous photo-oxidation (Table 1). Because Maraccini et al. (2016) reported shoulder durations averaging 462 and 890 minutes during the incubation of *E. coli* K12 and *E. coli* O157:H7, respectively, under simulated sunlight (400 $W \cdot m^{-2}$) in presence of natural photosensitizers, it is possible that the absence of significant decay was linked to the existence of shoulder log-linear kinetics. Nevertheless:

- Based on hourly sunlight data in Palmerston North, an incident sunlight intensity $\geq$ 400 $W \cdot m^{-2}$ was continuously reached for over 462 minutes on 98

days in 2016 (New Zealand National Institute for Water and Atmospheric Research Ltd), and it was never sustained for more than 890 minutes (at most, a 400 $W \cdot m^{-2}$ sunlight intensity was sustained for 600 minutes).

- Demory et al. (2018) computed the sunlight intensity history of a typical cell in a HRAP broth: based on this study, an *E. coli* cell normally circulating in a HRAP would never experience more than 3/8 of the incident sunlight intensity for the 3h displayed in the experiment.
- The duration of the shoulder period typically increases with decreasing sunlight intensity (Giannakis et al., 2015).

Therefore, although the low decay rates recorded during laboratory assays in the probable presence of exogenous photo-sensitizers (Fig S12-2) may be linked to the measurement of *E. coli* decay in the shoulder period of first order kinetics, exogenous photo-oxidation is unlikely to become significant in HRAP broth attenuating light considering the results listed above. Paired two samples t-test for the null hypothesis that *E. coli* decay in the probable presence and in the absence of photosensitizers did not differ (with the alternative hypothesis that *E. coli* decay was higher in probable presence of photosensitizers, one tailed test) did not reject the null hypothesis ($p = 0.584$, $N = 9$). For this hypothesis test, averaged data was used when two decay rates were measured in the same conditions.

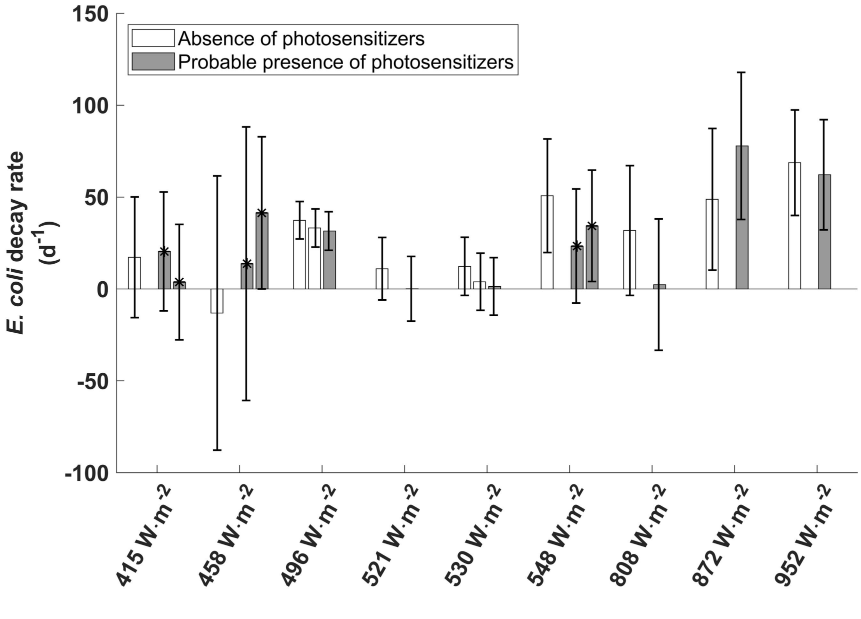

Fig. S12-2. ***E. coli*** **decay rate under natural sunlight in probable presence of photosensitizers (HRAP, no symbol, or wastewater, *, filtrates) or absence (pure water) of exogenous photosensitizers at neutral pH (laboratory assays).** The errors associated with these experiments were high due to the combination of relatively low decay rates recorded over short periods (not shown).

Demory, D., Combe, C., Hartmann, P., Talec, A., Pruvost, E., Hamouda, R., Souillé, F., Lamare, P.O., Bristeau, M.O., Sainte-Marie, J., Rabouille, S., Mairet, F., Sciandra, A., Bernard, O., 2018. How do microalgae perceive light in a high-rate pond? Towards more realistic Lagrangian experiments. R. Soc. Open Sci. 5. https://doi.org/10.1098/rsos.180523

Giannakis, S., Darakas, E., Escalas-Cañellas, A., Pulgarin, C., 2015. Solar disinfection modeling and post-irradiation response of Escherichia coli in wastewater. Chem. Eng. J. 281, 588–598. https://doi.org/10.1016/j.cej.2015.06.077

Maraccini, P.A., Wenk, J., Boehm, A.B., 2016. Exogenous indirect photoinactivation of bacterial pathogens and indicators in water with natural and synthetic photosensitizers in simulated sunlight with reduced UVB. J. Appl. Microbiol. 121, 587–597. https://doi.org/10.1111/jam.13183
