# Supplementary material for: Study from microcosms and mesocosms reveals Escherichia coli removal in high rate algae ponds during domestic wastewater treatment is primarily caused by dark decay
Source: PLoS One. 2022 Mar 17;17(3):e0265576. doi: 10.1371/journal.pone.0265576 (PMC8929646; doi:10.1371/journal.pone.0265576)
Supplement: S13 Appendix — (PDF) [file pone.0265576.s013.pdf]

S13 *E. coli* decay during bench assays: relationship with environmental parameters

E. coli decay rates from bench assays were calculated from two consecutive samplings as done when analysing the data from laboratory assays conducted under sunlight (S4). The decay rates calculated were analysed in light of sunlight intensity, pH, DO concentration, and temperature. Since pH, DO concentration, and temperature varied over time, the values of these parameters were averaged between two sampling events for numerical analysis (this averaging is acceptable because the variations experienced over the short interval between two consecutive samplings were small).

In the following, decay rates calculated from tests performed in the dark and under sunlight are presented, followed by a statistical analysis to identify which parameters significantly influenced *E. coli* survival during bench assays.

***E. coli* decay in darkness:** As shown in Fig S13-1, *E. coli* decay rate in darkness ranged from 13.5 d^{-1} (pH 7.3, 22.6 mg DO·L^{-1}, 34.3 °C) to 166 d^{-1} (pH 10.7, 20.0 mg DO·L^{-1}, 31.0 °C). The highest decay rates reported were all achieved at pH > 10 further confirming alkaline-pH induced toxicity likely cause significant *E. coli* decay in HRAP broth. Although this conclusion is limited by the low number of samples at alkaline pH, in three occurrences at pH higher than 10.4, no viable *E. coli* cells were found in any of the samples collected after inoculation, meaning no rates could be calculated. Based on the data shown in Fig S13-1, neither DO concentration nor temperature seem to influence *E. coli* decay. Temperature being uncontrolled during bench assays, the range

of temperatures recorded was small (28.1 – 34.3 °C) meaning any effect could have been hidden by other parameters variations or measurement uncertainty.

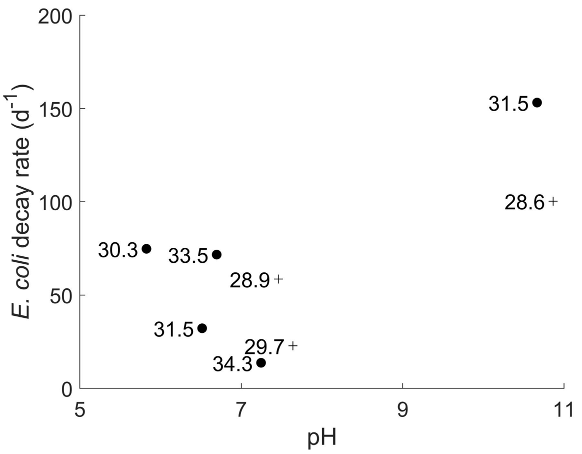

Fig. S13-1. Decay rates during bench assays in darkness. Closed circles (●) and crosses (+) represent rates recorded when DO > 8 $mg.L^{-1}$ and < 2 $mg.L^{-1}$, respectively. Labels show broth temperature (°C).

***E. coli* decay under sunlight:** The dataset of *E. coli* decay rates recorded during bench assays under sunlight is shown below grouped for high or low pH (Fig S13-3), and high or low DO concentration (Fig S13-4). As can be seen, pH and DO concentration likely increased *E. coli* decay rate under sunlight.

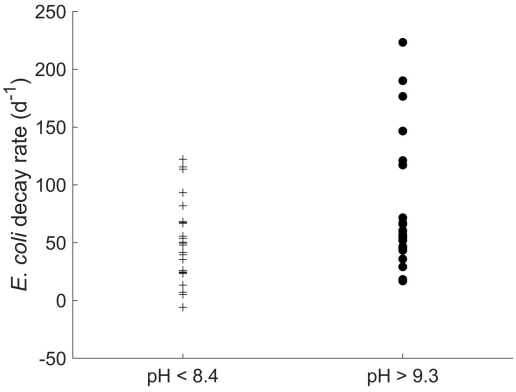

Fig. S13-2. ***E. coli*** **decay rate under sunlight measured during bench assays grouped for pH < 8.4 and pH > 9.3**

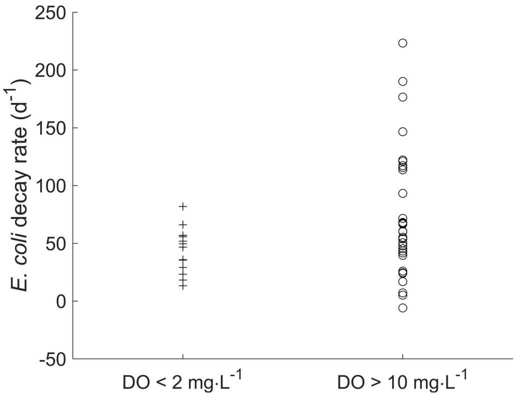

Fig. S13-3. ***E. coli*** **decay rate under sunlight measured during bench assays grouped for DO < 2 mg·L^{-1} and DO > 10 mg·L^{-1}**

While cross-comparison of simultaneous experiments had concluded pH was associated to higher log removal values (LRVs) under sunlight exposition, high DO concentrations were not associated with higher LRVs under sunlight (see main manuscript). This potential correlation was further tested through statistical analysis as described below.

Statistical analysis

Statistical hypothesis testing was performed to verify the hypotheses that *E. coli* decay was significantly influenced by 1) sunlight, 2) pH, 3) DO concentration, 4) pH under sunlight, 5) pH in the dark, 6) DO concentration under sunlight, and 7) DO concentration in the dark were tested. To achieve so, *E. coli* decay rate dataset was grouped in the following pairs of samples (respectively):

1) sunlight *versus* darkness,
2) high pH (> 9.3) *versus* low pH (< 8.4),
3) high DO concentration (> 10 $mg \cdot L^{-1}$) *versus* low DO concentration (< 2 $mg \cdot L^{-1}$),
4) high pH under sunlight *versus* low pH under sunlight,
5) high pH in the dark *versus* low pH in the dark,
6) high DO concentration under sunlight *versus* low DO concentration under sunlight,
7) high DO concentration in the dark *versus* low DO concentration in the dark.

Because temperature was not controlled, it could not be categorised and no hypothesis for the effect of temperature on *E. coli* decay was tested.

All numerical analysis were performed using Matlab® R2019a, Mathworks, Massachusetts, USA.

Prior to performing hypothesis testing, homogeneity of the non-tested parameters between each pair of samples tested was verified: for instance, homogeneity of DO concentration, sunlight intensity, and temperature between the sample of high pH and the sample of low pH was verified. Homogeneity was verified using Kruskal-Wallis hypothesis testing since environmental parameters distributions within samples were not

normal and unbalanced. The results from homogeneity testing (presented as the p-value of Kruskal-Wallis tests) and associated conclusions for the validity of further hypothesis testing are summarized in Table S13-1.

Table S13-1. Description of the samples defined for further hypothesis testing and summary of results from homogeneity testing for parameters of interest

Sample 1	N[1]	Sample 2	N[1]	Subset tested	Parameter homogeneity tested	Homogeneity test pvalue (Krustall-Wallis)[2]	Comment on homogeneity hypothesis testing
Low pH	30	High pH	24	All data	Sunlit	0.1289	Hypothesis testing **valid**
					DO	0.6383	
					Temperature	0.4594	
Low pH, Sunlit sample	24	High pH, Sunlit	22	Sunlit	Sun	0.0045	Hypothesis testing **invalid**: **Higher** sun with **lower** pH
					DO	0.775	
					Temperature	0.8345	
Low pH, Dark	6	High pH, Dark	2	Dark	Sun	NA	Hypothesis testing **invalid**: not enough data
					DO	NA	
					Temperature	NA	
Low DO	16	HighDO	38	All data	Sun	0.0527	Hypothesis testing **invalid**: **higher** temperature with **higher** DO
					pH	0.4543	
					Temperature	0.000914	
Low DO, Sunlit	13	HighDO, Sunlit	33	Sunlit	Sun	0.0378	Hypothesis testing **invalid**: **higher** temperature with **higher** DO **higher** sun with **higher** DO
					pH	0.7604	
					Temperature	0.0015	
Low DO, Dark	3	HighDO, Dark	5	Dark	Sun	NA	Hypothesis testing **invalid**: not enough data
					pH	NA	
					Temperature	NA	
Sunlit	46	Dark	8	All data	pH	0.1474	Hypothesis testing **invalid**: **higher** temperature in **sunlit** sample
					DO	0.3676	
					Temperature	0.0259	

[1] When a sample of less than 5 data points, the hypothesis testing was concluded to be too weak to be applicable.
[2] pvalue > 0.05 indicates the homogeneity hypothesis is accepted and that further hypothesis testing is not jeopardized by the distribution of the parameter. pvalue < 0.05 indicates the rejection of the null hypothesis meaning that the parameter tested has significantly different distributions on both cluster tested, and sample homogeneity hypothesis is not valid.

Since the data sub-samples had small size, unbalanced number of observations, and decay rate observations had unequal variances, two-sample ttest assuming unequal

variance (also known as Welch ttest) was preferred over ANOVA to test the potential influence of environmental parameters on *E. coli* decay rate. The null hypothesis postulated that the mean decay rates were equal between samples, the alternative hypothesis being that the samples of high pH (resp. high DO concentration, sunlight exposure) had a higher mean (one-tailed ttest). All hypothesis formulated earlier were tested regardless of the conclusion from homogeneity testing and results are shown in Table S13-2, but the consequences from homogeneity testing (Table S13-1) are discussed below.

Table S13-2. Summary from statistical analysis for testing the influence of environmental parameters on *E. coli* decay rate during bench assays

Null hypothesis tested (H0)	Alternative hypothesis (H1)	Sample 1	N	Sample 2	N	Comment on hypothesis testing[1]	Hypothesis testing pvalue (Welch ttest)[2]
High pH sample exhibit **equal** decay as low pH sample	High pH sample exhibit **higher** decay than low pH sample	Low pH	30	High pH	24	Valid	**0.0072**
		Low pH, Sunlit sample	24	High pH, Sunlit	22	Invalid	**0.025**
		Low pH, Dark	6	High pH, Dark	2	Invalid	0.082
High DO sample exhibit **equal** decay as low DO sample	High DO sample exhibit **higher** decay than low DO sample	Low DO	16	HighDO	38	Invalid	**0.0086**
		Low DO, Sunlit	13	HighDO, Sunlit	33	Invalid	**0.048**
		Low DO, Dark	3	HighDO, Dark	5	Invalid	0.402
Sunlit sample exhibit **equal** decay as dark sample	Sunlit sample exhibit **higher** decay than dark sample	Sunlit	46	Dark	8	Invalid	0.5278

[1] See Table S13-1.

[2] pvalue > 0.05 indicates the hypothesis of equality of decay rate between samples is accepted meaning the parameter tested has no influence on *E. coli* decay. pvalue < 0.05 indicates the rejection of the null hypothesis meaning that the parameter tested has a significant positive impact on *E. coli* decay.

The only statistical hypothesis testing which could formally be performed was the test for an influence of pH on *E. coli* decay rate on the full sample. This test validated the positive impact of pH on *E. coli* decay ($p = 0.0072$) as was concluded from cross comparison of bench reactors (main manuscript).

A statistically higher decay rate was also found on the high pH sub-sample compared with low pH sub-sample for the sunlit sample ($p = 0.025$) but this test was biased as the low pH sub-sample was associated to higher sunlight intensity than the high pH sub-sample. Nevertheless, increasing sunlight intensity was associated with increasing *E. coli* decay during laboratory assays, meaning the low pH sub-sample should have experienced higher decay rate due to sunlight intensity lack of homogeneity. Therefore, the statistically higher decay rate effectively associated to the high pH sub-sample should have been more significant if sunlight intensity had been homogeneously distributed on both sub-samples. It can realistically be concluded that high pH was also associated to significantly higher decay rate than neutral pH under sunlight.

When testing for a positive effect of DO concentration on *E. coli* decay both on the full sample and the sunlit sample, the data was biased by significantly higher temperature on the high DO concentration sub-samples. While a positive impact of DO concentration was reported for the full sample ($p = 0.0086$) and the sunlit sample ($p = 0.046$), no conclusions could be reached since higher temperature are expected to also favour higher decay. Cross comparison of bench assays concluded that DO concentration was unlikely to impact *E. coli* survival and that the high decay of *E. coli* under high DO

concentration was instead probably due to a combination of high pH and higher temperature.

Likewise, temperature biased the analysis when testing for a positive impact of sunlight on *E. coli* decay rate as the sunlit sub-sample experienced statistically higher temperatures than the dark samples ($p = 0.026$). Because temperature has likely a positive impact on *E. coli* decay rate, the absence of higher decay rate on the high sunlight sub-sample suggested by Welch ttest ($p = 0.5278$) despite higher temperature should have been confirmed if temperature had been homogeneously distributed on both sub-samples. It can realistically be concluded that *E. coli* decay under sunlight was not higher than in the dark.

Due to the low amount of data recorded in the dark, statistical tests performed on dark sub-samples were not significant enough to be discussed.

Statistical analysis confirmed with high confidence that pH had a positive impact on *E. coli* decay during bench assays, but that sunlight had little influence at high algal density. No conclusion could be reached for the impact of DO concentration due to a temperature bias in the data, nor for the specific impact of any parameter in the dark due to the low amount of data obtained (following higher than expected decay in the dark).
