# Supplementary material for: Study from microcosms and mesocosms reveals Escherichia coli removal in high rate algae ponds during domestic wastewater treatment is primarily caused by dark decay
Source: PLoS One. 2022 Mar 17;17(3):e0265576. doi: 10.1371/journal.pone.0265576 (PMC8929646; doi:10.1371/journal.pone.0265576)
Supplement: S14 Appendix — (PDF) [file pone.0265576.s014.pdf]

S14 *E. coli* decay due to sunlight direct damage in opaque broth: Model development

In relatively clear broth, the decay of *E. coli* in a volume exposed to uniform sunlight intensity I ($W \cdot m^{-2}$) could be described by a first order law of coefficient k_d (d^{-1}) expressed as:

$$k_d = 0.0624 \cdot I \tag{S14-1}$$

In a HRAP, sunlight attenuation is assumed to follow a Beer-Lambert law(Béchet et al., 2015) described as:

$$I(z) = I_0 . \exp(-\sigma . z) \tag{S14-2}$$

Where $I(z)$ is the light intensity at the depth z (m), σ (m^{-1}) is the light attenuation coefficient in the broth, and I_0 is the incident sunlight intensity at the broth surface ($W \cdot m^{-2}$).

Sunlight intensity is therefore considered uniform at depth z and *E. coli* decay at z can be calculated as:

$$k_d(z) = 0.0624 . I_0 . \exp(-\sigma . z) \tag{S14-3}$$

Assuming *E. coli* cell do not move vertically over a short time step δt, the variation of *E. coli* cells $C(z, t)$ during δt at depth z can then be determined as:

$$C(z, t + \delta t) = C(z, t). [1 - 0.0624. I_0. \exp(-\sigma. z)] \quad \textbf{(S14-4)}$$

In a well-mixed HRAP, the total cell count change over δt can be expressed as:

$$C(t + \delta t) = \frac{1}{V}. \int_{z=0}^{d} C(z, t + \delta t). S. dz \quad \textbf{(S14-5)}$$

Where $S.dz$ represents the volume between the depths z and $z + dz$, S (m^2) is the pond surface, and V the pond volume (m^3). This expression is integrated to:

$$C(t + \delta t) = C(t). [1 - \frac{0.0624. I_0}{\sigma. d}. \exp(-\sigma. d). \delta t] \quad \textbf{(S14-6)}$$

Equation S14-6 is analogous to a first order decay law in batch reactor at a rate k_d described by.

$$k_d = \frac{0.0624. I_0}{\sigma. d}. \exp(-\sigma. d) \quad \textbf{(S14-7)}$$

This decay rate was adopted to model the first order decay of *E. coli* due to direct sunlight exposition in the full HRAP broth during bench scale experiments.

Béchet, Q., Chambonnière, P., Shilton, A., Guizard, G., Guieysse, B., 2015. Algal productivity modeling: A step toward accurate assessments of full-scale algal cultivation. Biotechnol. Bioeng. 112. https://doi.org/10.1002/bit.25517
