# Supplementary material for: Study from microcosms and mesocosms reveals Escherichia coli removal in high rate algae ponds during domestic wastewater treatment is primarily caused by dark decay
Source: PLoS One. 2022 Mar 17;17(3):e0265576. doi: 10.1371/journal.pone.0265576 (PMC8929646; doi:10.1371/journal.pone.0265576)
Supplement: S15 Appendix — (PDF) [file pone.0265576.s015.pdf]

S15 Sensitivity analysis of *E. coli* decay rate modelling in HRAP broth

Model sensitivity was evaluated by individually varying the experimental input parameters (Table S5-1) from their average values by ± standard deviation. The best-fit model parameters were estimated each time as described in the main manuscript. As can be seen in Fig S15-1, model parameterization was highly sensitive to *E. coli* cell counts uncertainty. This uncertainty is unfortunately hard to reduce as all bacterial count methods are characterized by significant uncertainty.

The parameterization of k_{20}^{dark} and k_{20}^{pH} was relatively more sensitive to model inputs than the parameterization of θ^{dark} and θ^{pH}. The low sensitivity of θ^{dark} suggests that uncharacterized dark decay is likely to be temperature-independent ($\theta^{dark} \approx 1$), although testing over a wider range of temperature is still needed to confirm this.

The sunlight specific decay rate α exhibited little sensitivity to any of the tested parameters and it can be confidently concluded that sunlight direct damage had a negligible contribution to *E. coli* removal during bench scale experiments. Since all experiments were performed in November in New Zealand (i.e. at high algal and optical density), similar experiments conducted during winter (i.e. in clearer broth) may evidence a larger relative contribution of sunlight mediated disinfection.

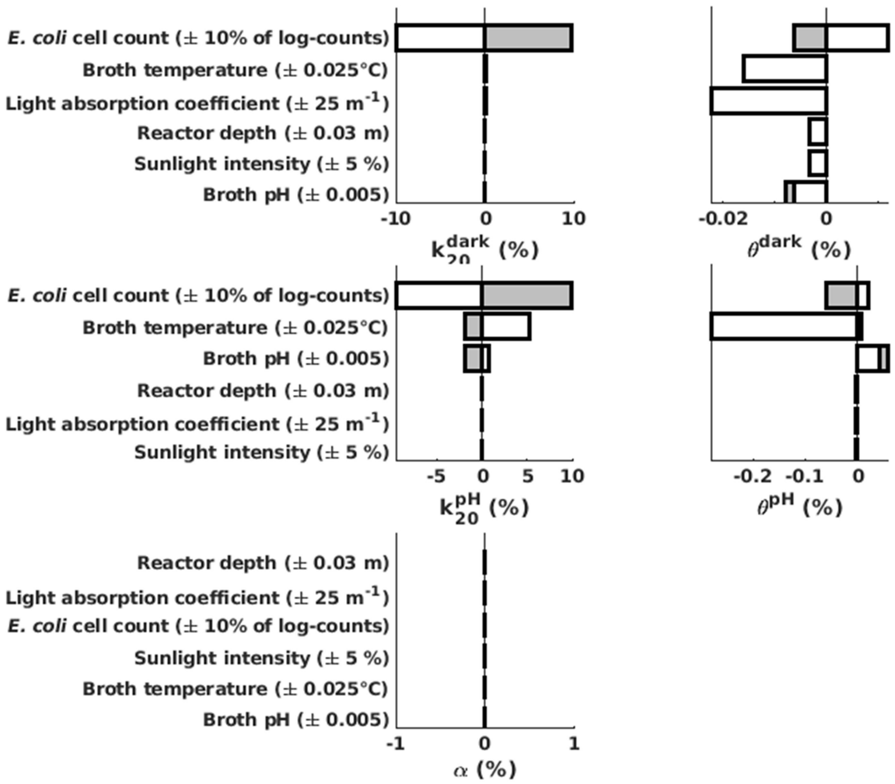

Fig. S15-1. Tornado diagram of model sensitivity to parameter and measurement uncertainties (outputs shown corresponds to the percentage of deviation from the best fit value, Table 2)
